# Involvement of Annexin A2 Expression and Apoptosis in Reverse Polarization of Invasive Micropapillary Carcinoma of the Breast

**DOI:** 10.1155/2020/9242305

**Published:** 2020-07-10

**Authors:** Kazumori Arai, Tomohiro Iwasaki, Chinatsu Tsuchiya, Akihiro Sonoda

**Affiliations:** ^1^Department of Pathology, Shizuoka General Hospital, 4-27-1 Kitaando, Aoi-ku, Shizuoka 420-0881, Japan; ^2^Department of Clinical Research, Shizuoka General Hospital, Shizuoka, Japan

## Abstract

Invasive micropapillary carcinoma (IMPC) is characterized by pseudopapillary tumor-cell clusters with a reverse polarity (RP) floating in lacunar spaces, with aggressive biological characteristics. The RP prevention is considered to inhibit IMPC, but its pathogenic mechanisms remain unclear. Annexin A2 (ANX A2), a cell-polarity protein, is known to be involved in lumenogenesis. ANX A2 expression is immunohistochemically examined, as well as both epithelial membrane antigen (EMA) and mucin-1 glycoprotein (MUC-1), the gold-standard markers for luminal differentiation, in the background tumor components of a case of IMPC. The following findings were noticed: (1) Apoptosis was scattered with peripheral apoptotic vacuolar change; (2) EMA and MUC-1 expressions were found, rimming the peripheral apoptotic vacuoles (including the contact surface with neighboring tumor cells), and these positions corresponded to the ones with a distinct ANX A2 positivity; and (3) partially detached tumor cells showed distinct positivity of three proteins at the stroma-facing surface, which is consistent with a RP. Taken together, frequent apoptosis in tumor cells with membranous accumulation of ANX A2 is considered to be indispensable for the reverse polarization of IMPC, and that secondary necrosis following apoptosis induces the cell-polarity disorder and creates detached tumor cells with a RP.

## 1. Introduction

Invasive micropapillary carcinoma (IMPC) is characterized by tumor-cell clusters devoid of fibrovascular cores, floating in lacunar spaces [1, 2]. This unique breast tumor was first defined as having an “exfoliative appearance” by Fisher et al. [3] and first proposed as “invasive micropapillary carcinoma” by Siriaunkgul and Tavassoli [1]. Since then, IMPC has been reported in various organs and known to have aggressive biological characteristics, such as lymphovascular invasion and node metastasis [4]. Furthermore, the immunohistochemical analysis has demonstrated that IMPC cells show a reverse polarity (RP), i.e., an inside-out growth pattern [2, 4, 5]. The RP is a major characteristic of IMPC, whereby the luminal surface of the tumor cells faces the stroma [2, 4, 5].

In the breast, pure IMPC is rare, accounting for approximately 0.9%–2% of invasive breast cancers [2], whereas IMPC is found as one of the tumor components associated with invasive carcinoma of no special type (IC-NST) [1, 2, 6, 7]. The intrinsic type of IMPC widely varies; however, approximately half of them are luminal A type (hormone receptor-positive and HER2/neu-negative) [8]. Generally, the prognosis of IC-NST of luminal A type is known to be better than that of other intrinsic types [2]. Nevertheless, the IMPC component has been reported to adversely affect the prognosis of breast cancer even if it is not dominant [7]. Whether a micropapillary (MP) phenotype of the breast is an independent prognostic factor remains debatable [2, 7–10]. However, IMPC also has been known to have a high proclivity to locoregional recurrence [9, 10]. The RP prevention is considered to inhibit the occurrence of MP component in breast cancer, but its pathogenic mechanisms remain unclear [6]. The MP morphological formation has been shown to occur in tumor cells with both high apoptosis and low proliferation in the ductal carcinoma in situ (DCIS) of the breast [11]. We believe that IMPC will be inhibited depending on an understanding of the protein(s) involved in the reverse polarization.

Annexins (ANXs) are a multigene family of calcium-related and membrane-binding proteins that show cell-specific expression [12]. Twelve human ANX subtypes (A1–A11 and A13) have been described, and each ANX is distributed differentially and has varied functions in cellular processes, such as calcium signaling, cytoskeletal organization, growth regulation, cell division, and apoptosis [12]. Annexin A2 (ANX A2), a 36 kDa protein, is involved in diverse cellular functions, such as cell motility/invasion, cell polarity, cell adhesion, and apoptosis, within the cytoplasm and plasma membranes [13]. Moreover, in glandular epithelial cells, this protein is involved in lumenogenesis, that is, the establishment of apical polarity [14, 15]. We hypothesized that ANX A2 may also be involved in a RP and encountered a case of IMPC that helped elucidate the reverse polarization in IC-NST. In this case report, we immunohistochemically examined the localization of ANX A2 and also investigated the expressions of both epithelial membrane antigen (EMA) and mucin-1 glycoprotein (MUC-1), the gold-standard markers for luminal differentiation [4, 5].

## 2. Case Presentation

**Figure 1:**
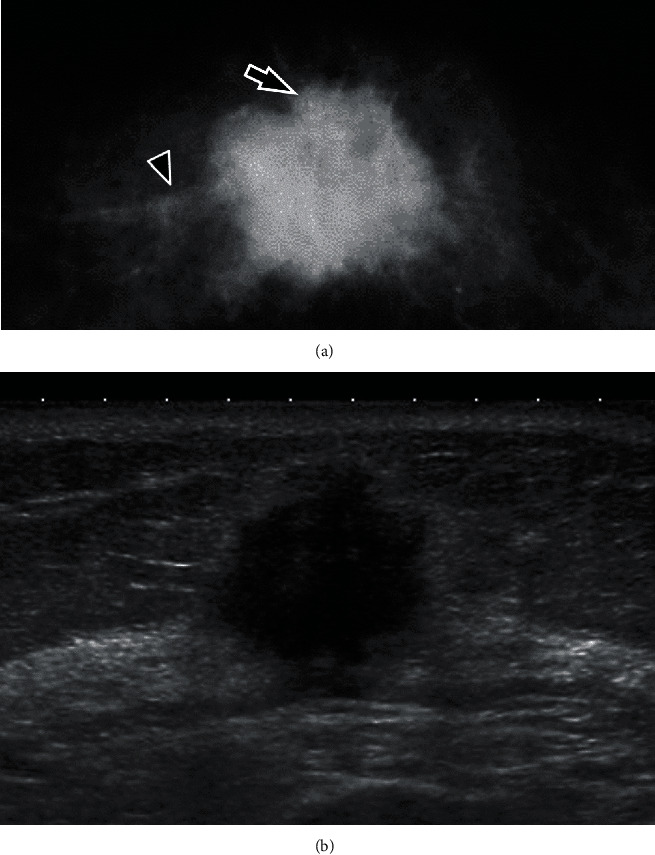
Mammography (a) and ultrasound (b) of the right breast. (a) Mammography shows a high-density mass with segmental microcalcifications (arrow) and specular borders (arrowhead). (b) Ultrasound shows an irregular hypoechoic mass with decreased posterior echo.

A 56-year-old woman presented with a dimpling breast lump for 6 months. She had no family history of breast and other cancers. Physical examination revealed a discrete mass at the middle inner quadrant of the right breast. Mammography showed a high-density mass of 2.3 cm in diameter with segmental microcalcifications and specular borders that caused slight skin retraction (Figure 1(a)). Ultrasound demonstrated an ill-circumscribed and hypoechoic mass of 2.2 × 2.1 cm in size with decreased posterior echo (Figure 1(b)). Enhanced computed tomography suggested multiple metastases to the right axillary lymph nodes. No metastasis to the other organs was noted. Core needle biopsy revealed an IC-NST with IMPC components. Furthermore, fine needle aspiration cytology from the right axillary lymph node detected adenocarcinoma, indicating metastasis. Radical mastectomy with lymph node dissection was performed about 1.5 months after her first visit. Postoperatively, the patient received various chemotherapy treatment for 9 months, such as 4 cycles of epirubicin and cyclophosphamide, 4 cycles of docetaxel, and 25 cycles of radiation. Then, antihormonal therapy with letrozole has been administered to date, without signs of recurrence during the follow-up period of 14 months.

To examine reverse polarization, the histopathology of the background IC-NST that is regarded as the origin of IMPC components was examined. Immunohistochemistry was performed with serial tissue sections, using Leica Bond-Max (Leica Biosystems, Australia). The characterization and staining protocol of each antibody for ANX A2 (catalogue number, 610068) and MUC-1 (catalogue number, NCL-MUC-1) have been described previously [16, 17]. Mouse anti-human EMA antibody (clone, GP 1.4; catalogue number, NCL-EMA; Leica Microsystems, UK) was used at a dilution ratio of 1 : 500, after a 10 min heat antigen retrieval with a citrate-based solution (pH 6.0). To detect apoptosis, the mouse monoclonal antibody M30 CytoDEATH (clone, M30; catalogue number, 12 140 322 001; Roche Diagnostics, Germany) was used at a 1 : 100 dilution ratio, after a 20 min heat antigen retrieval with an EDTA-based solution (pH 9.0). This antibody detects caspase cleavage products of cytokeratin subtype 18 in apoptotic glandular epithelial cells, including adenocarcinoma cells, and is considered to be more specific and reliable than the TUNEL assay [18].

## 3. Pathological Findings

**Figure 2:**
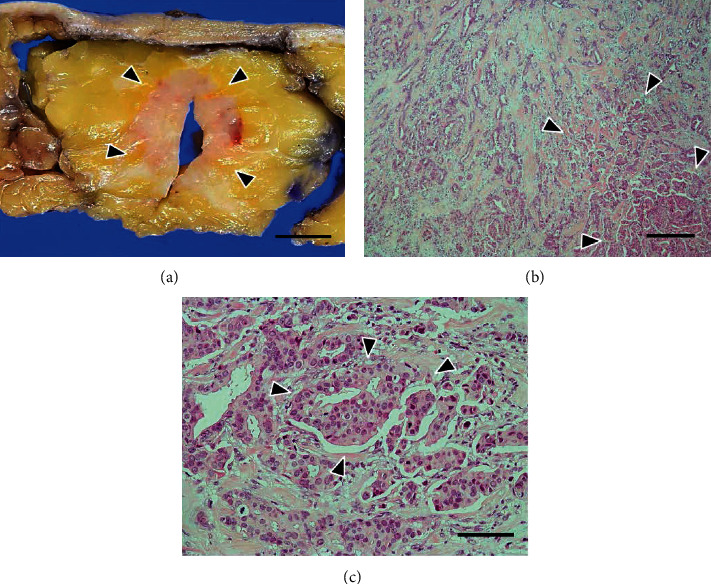
Routine pathological findings of the right breast cancer. (a) Gross view shows an ill-defined solid tumor (surrounded by arrowheads). Scale bar: 1 cm. (b) Invasive micropapillary carcinoma (IMPC) components (surrounded by arrowheads) are seen with the background of tubule-forming invasive carcinoma. Hematoxylin and eosin (H&E) stain, scale bar: 400 *μ*m. (c) IMPC components are mixed in the background tumor, with the transition in histology (surrounded by arrowheads). H&E stain, scale bar: 100 *μ*m.

**Figure 3:**
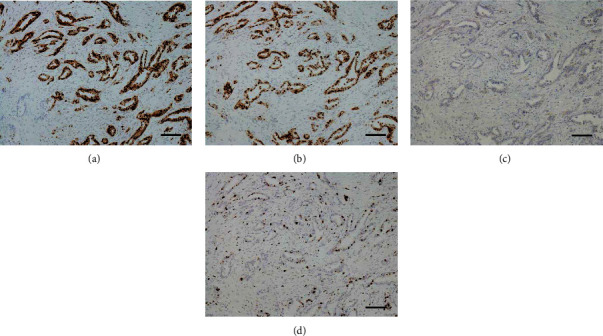
Routine immunohistochemical examination of the right breast cancer. (a) Majority of tumor cells represent a strong immunoreactivity for estrogen receptor (ER). ER immunostain. (b) Most tumor cells show a positive reaction for progesterone receptor (PGR). PGR immunostain. (c) Only a weak and heterogeneous positivity is observed in <10% of tumor cells, which are known to be negative at HercepTest score 0. HER2/neu-immunostain. (d) Approximately 30% of tumor cells are positive for Ki-67. Ki-67 immunostain. The scale bars indicate 100 *μ*m.

Macroscopic examination showed that the tumor was a solid mass of 25 × 24 mm in size, with an irregular margin (Figure 2(a)). Microscopic findings also revealed that majority of the tumor exhibited IC-NST with tubular formation (Figure 2(b)), and IMPC components were mixed during the transition in histology (Figures 2(b) and 2(c)). The total amount of tumor-cell clusters with typical MP morphology was approximately 20% of the tumor components. The tumor showed a histological grade of 2 (tubular formation, 2; nuclear atypia, 3; and mitosis, 1) [2]. Immunohistochemically, over 90% of the tumor cells tested positive for estrogen and progesterone receptors (estrogen receptor: clone, 6F11; catalogue number, NCL-L-ER-6F11; progesterone receptor: clone, 16; catalogue number, NCL-L-PGR-312; Leica Microsystems), respectively (Figures 3(a) and 3(b)), but negative at score 0 for HercepTest™ (catalogue number, SK001; DakoCytomation, Denmark) (Figure 3(c)). Based on these immunohistochemical results, the intrinsic type of the tumor under study corresponded to luminal A type [2]. The Ki-67 (clone, MIB-1; catalogue number, M 7240; DAKO) index was relatively low, approximately 30% (Figure 3(d)) [10]. The tumor revealed lymphatic invasion, and a total of 9 node metastases were detected during the level I axillary dissection.

### 3.1. Histopathological Findings of the Background IC-NST

**Figure 4:**
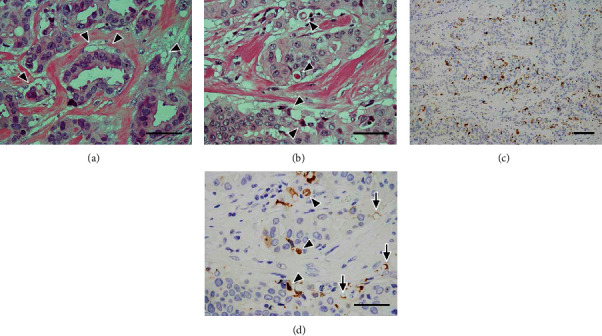
Multiple apoptosis in the right breast cancer I. (a) Concentrated vacuoles are partially or circumferentially found at the periphery of each component in the background tumor (arrowheads). H&E stain, scale bar: 50 *μ*m. (b) Apoptotic bodies and cells are scattered in the peripheral vacuoles (arrowheads), and inflammatory cell infiltration is inconspicuous. H&E stain, scale bar: 50 *μ*m. (c) Positive reactions are widespread mainly at the periphery of the background tumor components. M30 CytoDEATH immunostain, scale bar: 100 *μ*m. (d) Image corresponding to (b). Apoptotic bodies and cells represent immunoreactivity (arrowheads), and the positivity is also observed at the rim of the peripheral vacuoles (arrows). M30 CytoDEATH immunostain, scale bar: 50 *μ*m.

**Figure 5:**
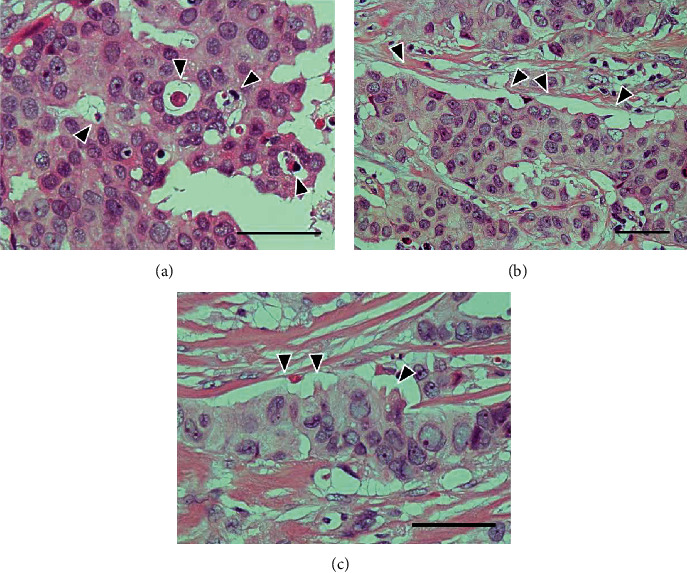
Multiple apoptosis in the right breast cancer II. (a) Apoptotic bodies are also observed inside the tumor component, forming pseudolumens (arrowheads). H&E stain. (b) Clear spaces without septa are found, considering partial tumor-cell detachment from the stroma (arrowheads). H&E stain. (c) Some of detached tumor cells show serrated peripheral borders (arrowheads). H&E stain. The scale bars indicate 50 *μ*m.

Concentrated vacuoles were partially or circumferentially found at the periphery of each tumor component (Figure 4(a)). In some vacuoles, apoptotic bodies and cells were found (Figure 4(b)). Regarding immunohistochemistry for M30 CytoDEATH, positive reactions were widespread mainly at the periphery of the background tumor components (Figure 4(c)) and corresponded to apoptotic bodies and cells (Figure 4(d)). Furthermore, the positive reactions were observed at the rim of the peripheral vacuoles (Figure 4(d)). Apoptotic bodies and cells were also observed inside the tumor-cell nests, and pseudolumens were scattered (Figure 5(a)). In some tumor components, clear spaces without septa were found, considering partial tumor-cell detachment from the stroma (Figure 5(b)). Moreover, some of the detached tumor cells showed serrated peripheral borders (Figure 5(c)). Inflammatory cell infiltration was inconspicuous in the tumor components (Figures 4 and 5).

### 3.2. Immunohistochemical Findings of the Background IC-NST

**Figure 6:**
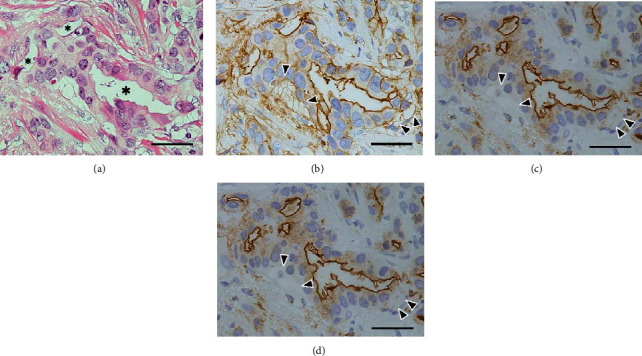
Immunohistochemistry of the background tumor components I. (a) An irregular-shaped tubular component with scattered peripheral vacuoles is seen. Asterisks indicate lumens. H&E stain. (b) Luminal surface shows a clear positivity, whereas the contact surface membranes represent a weak positivity. A weak positivity is also seen, rimming the individual peripheral vacuoles (arrowheads). Annexin A2 (ANX A2) immunostain. (c) Luminal surface shows a distinct positive reaction, but neither contact surface membranes nor the rims of peripheral vacuoles represent a clear positivity (arrowheads). Epithelial membrane antigen (EMA) immunostain. (d) The immunoreactivity similar to that of (c) is detected (arrowheads). Mucin-1 glycoprotein (MUC-1) immunostain. The scale bars indicate 50 *μ*m.

**Figure 7:**
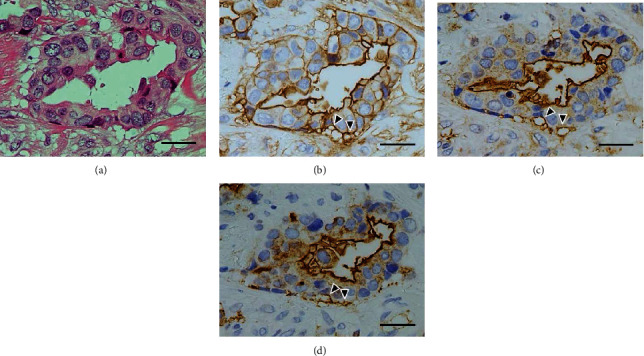
Immunohistochemistry of the background tumor components II. (a) A tubular component with scattered peripheral vacuoles is detected. H&E stain. (b) Luminal surface shows a distinct positivity, and a strong positivity is also observed at some rims of peripheral vacuoles, including the contact surface with the neighboring tumor cells (arrowheads). ANX A2 immunostain. (c) Luminal surface shows a distinct positivity, and a clear positivity is also found at some rims of the peripheral vacuoles with the strong ANX A2-positivity (arrowheads). EMA immunostain. (d) The immunoreactivity similar to that of (c) is seen (arrowheads). MUC-1 immunostain. The scale bars indicate 25 *μ*m.

**Figure 8:**
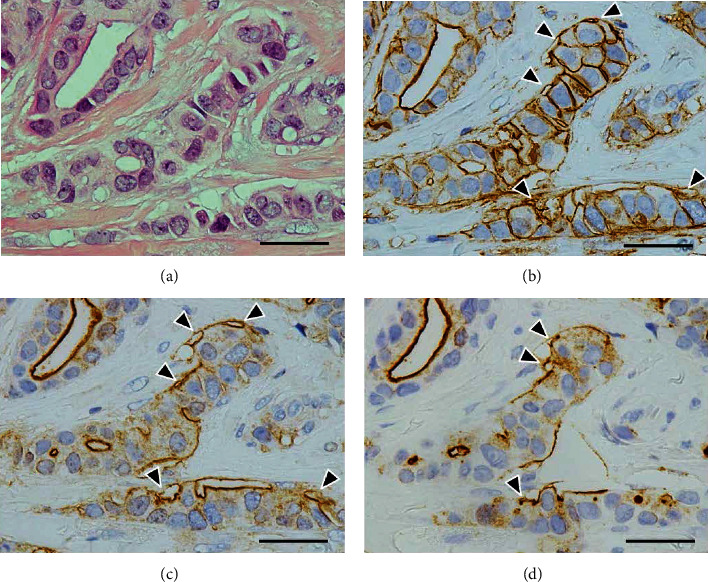
Immunohistochemistry of the background tumor components III. (a) Tumor-cell nests with peripheral vacuoles are seen. H&E stain. (b) A strong positivity is found, rimming some of peripheral vacuoles (arrowheads), and is also observed at the intercellular membranes between the neighboring tumor cells. Luminal surface also shows a distinct positivity. ANX A2 immunostain. (c) A distinct positivity is also detected at the rims of the peripheral vacuoles with strong ANX A2-positivity (arrowheads). EMA immunostain. (d) The immunoreactivity similar to that of (c) is detected (arrowheads). MUC-1 immunostain. The scale bars indicate 50 *μ*m.

**Figure 9:**
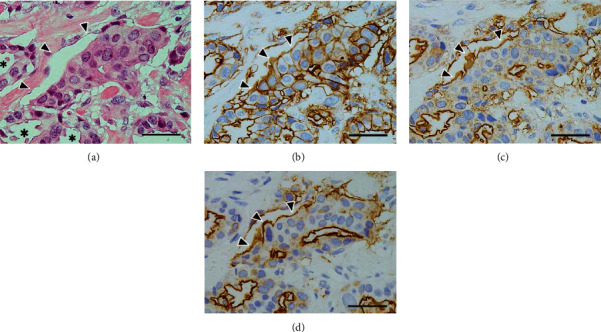
Immunohistochemistry of the tumor component showing partial detachment. (a) A cleft-like space without septa is found (arrowheads). Asterisks indicate lumens. H&E stain. (b) A stroma-facing surface of detached tumor cells shows a clear positivity (arrowheads). Moreover, the luminal surface, some rims of vacuoles, and intercellular membranes of the neighboring tumor cells show a distinct positivity. ANX A2 immunostain. (c) A distinct positivity is also seen at the stroma-facing surface of partially detached tumor cells, similarly to that of (b) (arrowheads). EMA immunostain. (d) The immunoreactivity similar to that of (c) is detected (arrowheads). MUC-1 immunostain. The scale bars indicate 50 *μ*m.

**Figure 10:**
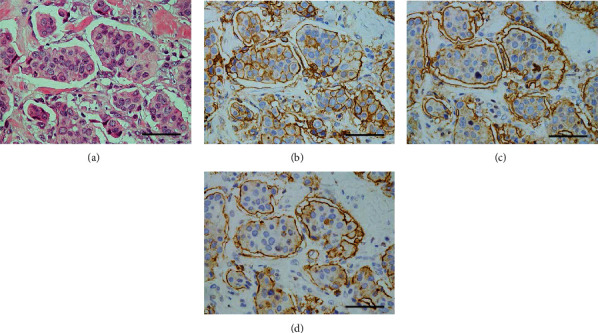
Immunohistochemistry of invasive micropapillary components. (a) Small tumor-cell clusters devoid of fibrovascular cores are seen, floating in lacunar spaces. H&E stain. (b) The rims of tumor-cell clusters show a circumferential linear positivity. ANX A2 immunostain. (c) A distinct positivity is circumferentially found at the rims of the clusters. EMA immunostain. (d) The immunoreactivity similar to that of (c) is detected. MUC-1 immunostain. The scale bars indicate 50 *μ*m.

Luminal surface constantly showed a distinct positivity for all three antigens: ANX A2, EMA, and MUC-1 (Figures 6–9). In contrast, several contact surface membranes represented a weak positivity for ANX A2 but were negative for EMA and MUC-1 (Figure 6). On the contrary, the immunoreactivity of peripheral vacuoles was significant, regardless of the size and numbers of vacuoles. In vacuoles with weak positivity for ANX A2, the distinct positivity for neither EMA nor MUC-1 could be detected (Figure 6). Conversely, in vacuoles with clear positivity for ANX A2, the distinct positivity for both EMA and MUC-1 was detected at the corresponding positions to the ANX A2 positivity. Furthermore, positive reactions for three antigens were found, rimming the individual vacuoles, including the contact surface with neighboring tumor cells (Figures 7 and 8). These reactions represented a shadow-like appearance of the cells leaving only the surface membrane, suggesting that the vacuoles are derived from apoptotic tumor cells. The ANX A2 positivity was also faintly observed even at the rims of the vacuoles in the sites without distinct positivity for both EMA and MUC-1 (Figure 6). The above clear positivity for ANX A2 was also observed in the intercellular membranes between tumor cells neighboring the vacuoles; however, the distinct positivity for neither EMA nor MUC-1 could be detected at the corresponding positions (Figure 8). In partially detached tumor cells, positive reactions for three antigens were found at the stroma-facing surface, representing a partial RP (Figure 9). In IMPC, the rims of the tumor-cell clusters showed the circumferential staining for three antigens (Figure 10).

## 4. Discussion

To our knowledge, apoptosis has been reported to be associated with completed MP components [19–21], whereas no reports have demonstrated the involvement of apoptosis in the process of MP morphology formation in invasive breast cancer. In the tumor under study, the background IC-NST showed histological findings consistent with those suggested in a previous report on DCIS [11]. The immunopositivity for M30 CytoDEATH at the rim of the vacuoles suggests that these vacuoles are shadows of apoptotic tumor cells. We consider that frequent apoptosis is also the initial step of MP morphology formation in the IMPC of the breast [11].

ANX A2 has been known to be deposited in the cell membranes that become a lumen in the future and accumulated in the luminal surface, being combined with apical polarity protein complex [14, 15]. In the present tumor, ANX A2 immunolocalization in the background IC-NST was consistent with the above ANX A2 dynamics [14, 15, 22]. Generally, lumenogenesis occurs between cells that contact each other [14, 15, 23]. Therefore, the distinct positivity for ANX A2 at the contact surface between peripheral vacuoles and neighboring tumor cells indicates ANX A2 accumulation on the surface membranes of both apoptotic and neighboring tumor cells [14, 15, 23–25]. In other words, the vacuoles are considered as the remaining cell membranes of the former tumor cells that were supposed to form the lumen in collaboration with the neighboring tumor cells [14, 15, 23–25]. Frequent apoptosis in the tumor cells with membranous ANX A2 accumulation is considered to be indispensable for reverse polarization [11, 14, 15, 23–25].

In some cancers, ANX A2 has been reported to inhibit tumor-cell apoptosis [26]. In the tumor under study, the cause of widespread apoptosis in tumor cells expressing ANX A2 remains unclear. In IMPC, the inhibitory function of ANX A2 on apoptosis may be impaired.

Secondary necrosis following apoptosis disappears even in the remaining cell membranes [24, 25]. Secondary necrosis of the apoptotic tumor cells with luminal differentiation is considered to partially create detached tumor cells with a RP [11, 14, 15, 23–25]. The partial RP is suggested to be a part of the IMPC spectrum in breast cancer [27, 28], and serrated peripheral borders shown in detached tumor cells are known as one of the characteristics of IMPC [29]. The MP morphology is assumed to be a unique tumor-cell condition in which those with an apical polarity could form the luminal surface facing the stroma [14, 15, 24, 25, 27, 28]. Whether lacunar space of IMPC is a shrinkage artifact or not remains controversial [1, 2, 27, 28]. However, several investigators have suggested that the space is not just an artifact but is intrinsically related to the biological features of IMPC [27, 28]. The lacunar space is also considered to significantly originate from secondary necrosis [24, 25, 27, 28].

Cell-stroma adhesion is known to be involved in both creation and maintenance of an apical–basal polarity of the cell in cooperation with cell–cell contacts [14, 15, 23]. Accordingly, both frequent apoptosis and secondary necrosis are considered to disturb the apical–basal polarity of the tumor cells everywhere, resulting in a condition resembling to partially polarized structures with dysplastic features [23–25]. In other words, a focal RP is likely to occur everywhere at the background IC-NST due to frequent apoptosis [14, 15, 23–25, 27, 28]. In detached tumor cells, the basal polarity is supposed to be impaired by loss of cell-stroma adhesion [23, 30]. Therefore, even detached tumor cells without luminal differentiation might acquire an apical polarity on the stroma-facing surface, under the effects of cell–cell contacts with the neighboring tumor cells with an apical polarity [23].

At the intercellular membranes between the tumor cells and neighboring peripheral vacuoles, a discrepancy in immunoreactivity was observed between ANX A2 and both EMA and MUC-1. If findings at those sites suggest the preluminal stage, that is, a preapical patch or a stage before its formation [14, 15, 23], neither EMA nor MUC-1 may possibly be detected in cell membranes with distinct ANX A2 positivity. Further examinations are needed to confirm this consideration.

Some investigators have reported that MUC-1 might promote tumor-cell detachment and MP morphology formation in IMAC [4, 28, 31]. However, in this tumor, the apoptotic peripheral vacuoles, as tumor-cell detachment preparations, were scattered, regardless of MUC-1 immunoreactivity. We consider that the MUC-1 involvement in the initial step of the MP morphology formation is lower than that of frequent apoptosis. Rather, MUC-1 might contribute to preventing the surface membrane of detached tumor cells from reattaching to the stroma [32].

In conclusion, the following findings were notable for reverse polarization in IMPC: (1) frequent apoptosis in the lumenogenic tumor cells with a membranous accumulation of ANX A2; (2) partially detached tumor cells with a RP, which are derived from secondary necrosis of apoptotic tumor cells with luminal differentiation. This study had some limitations because only one case was examined. Further investigations are needed to confirm our suppositions.
